# Prevalence and Morphological Characteristics of Taurodontism in Permanent First and Second Molars in a Peruvian CBCT Sample

**DOI:** 10.3390/dj14060357

**Published:** 2026-06-09

**Authors:** Estrellita Lisbeth Fiestas Purizaca, Lizbeth Marilen Yangua Vicente, Catherin Angélica Ruiz-Cisneros, Paul Martín Herrera-Plasencia

**Affiliations:** Facultad de Ciencias de la Salud, Escuela Profesional de Estomatología, Universidad César Vallejo, Piura 20009, Peru; estrellafiestas02@gmail.com (E.L.F.P.); yanguavicente48@gmail.com (L.M.Y.V.); cruizci@ucvvirtual.edu.pe (C.A.R.-C.)

**Keywords:** anatomical variation, pulp chamber, molars, maxilla, cone-beam computed tomography

## Abstract

**Introduction/Objective:** Taurodontism is an anatomical variation characterized by an increase in the pulp chamber, apical displacement of the chamber floor, and a relative reduction in root length. The objective was to determine the prevalence and morphological characteristics of taurodontism in permanent first and second molars within a Peruvian sample evaluated using cone-beam computed tomography (CBCT). **Materials and Methods:** An observational, cross-sectional, and retrospective study was conducted based on 382 CBCT scans of patients aged 18 to 59 years, acquired between 2022 and 2024. The prevalence was estimated at the patient/CBCT level, defined as the presence of at least one taurodontic permanent first or second molar, and at the tooth level, considering the present and evaluable molars. The association with sex and age group was evaluated using chi-square, odds ratios, and logistic regression adjusted for sex, age, and the number of evaluable molars. **Results:** Taurodontism was identified in 266/382 CBCT scans, with a patient/CBCT prevalence of 69.6%. In the adjusted model, an association was observed with the female sex (aOR = 1.73; 95% CI: 1.10–2.73; *p* = 0.018) and the 18–29 age group (aOR = 2.34; 95% CI: 1.45–3.77; *p* < 0.001). At the tooth level, 692/2472 evaluable molars were taurodontic (28.0%). Among the taurodontic molars, 502/692 were located in the maxilla (72.5%) and 190/692 in the mandible (27.5%). Hypotaurodontism was the predominant subtype (594/692; 85.8%). **Conclusions:** Molar taurodontism was frequent at the patient/CBCT level in this selected sample; however, its prevalence at the tooth level was lower. The associations with sex and age should be interpreted with caution.

## 1. Introduction

Dental anatomical variations are frequently observed in radiographic studies [1]; these can affect both the crown and the roots, resulting in changes in their shape, size, or morphology that may influence the planning of dental treatment [2]. They have been linked to various factors, including hereditary, environmental, and systemic factors, as well as to abnormalities occurring during odontogenic development [1,3]. Among the dental anomalies described, taurodontism is characterized by a morphology marked by vertical expansion of the pulp chamber, with apical displacement of the chamber floor and the furcation, resulting in an elongated tooth body and relatively short roots [4,5]. From a clinical standpoint, these variations can affect the location and configuration of the root canals, increasing the complexity of endodontic examination, instrumentation, and obturation, as well as certain orthodontic and surgical procedures, which may influence the therapeutic prognosis [6,7].

The term taurodontism was first described by Sir Arthur Keith in 1913 [8] and may be associated with abnormalities in the invagination of Hertwig’s epithelial sheath, which is responsible for root morphogenesis [6,9]. It can occur in isolation or in association with genetic syndromes such as Klinefelter, Down, osteogenesis imperfecta, and Torg-Winchester, highlighting its etiopathogenic diversity and possible relationship with systemic factors [10].

In this context, the accurate identification of this anomaly is essential for its proper morphological characterization. Diagnosis based on two-dimensional radiographs may underestimate the actual morphology of taurodontism due to the overlap of structures and spatial limitations; in contrast, cone-beam computed tomography (CBCT) allows for three-dimensional evaluation using multiplanar sections, with higher resolution and greater accuracy in measuring dental structures, which facilitates more precise and reproducible identification and classification [11,12,13].

Under this diagnostic approach, various epidemiological studies have reported variations in the prevalence of taurodontism across different populations. In China, a study found that 29.14% of the patients evaluated had taurodontism, most frequently in the maxilla (9.06%) and in the upper second molars (25.18%), with the hypotaurodontic subtype being the most common (60.39%) [7]. In Saudi Arabia, a study reported a prevalence of 8%, primarily among individuals aged 21 to 40, with hypotaurodontism being the most common subtype (67.6%) [14]. For its part, in Pakistan, taurodontism was found in 9.16% of the cases evaluated, with a higher frequency in the maxilla (6.66%) [15]. Likewise, in Iran, a prevalence of 20.9% was reported, particularly in the upper jaw (35%), with a predominance of hypotaurodontism (75%) [16]. In Turkey, it was found that taurodontic teeth were most frequently located in the upper second molars (12.72%), with a higher proportion among females [17].

Taurodontism poses a clinical challenge due to its anatomical complexity and its implications for various dental procedures. However, there remains an epidemiological gap regarding its prevalence and morphological characteristics in Latin America, particularly in Peruvian CBCT-based samples. Determining its frequency and anatomical distribution is important for more precise clinical planning, optimizing the indication and interpretation of imagenological techniques, and reducing the risk of complications associated with dental management. In this context, the objective of this study was to determine the prevalence and morphological characteristics of taurodontism in permanent first and second molars in a Peruvian CBCT sample.

## 2. Materials and Methods

### 2.1. Study Design and Ethical Approval

An observational, retrospective, cross-sectional study was conducted, approved by the Research Ethics Committee of the School of Stomatology at César Vallejo University, Piura, Peru (Resolution No. 00008-2024/CEI-EST; 4 June 2024). The study was based on a systematic analysis of cone-beam computed tomography (CBCT) scans acquired at a private radiology center in Piura, Peru, from patients referred for routine dental evaluation between 2022 and 2024. The images were anonymized prior to analysis.

### 2.2. Sample and Eligibility Criteria

Cone-beam computed tomography (CBCT) scans of patients aged 18 to 59 years were included, provided they demonstrated adequate diagnostic quality and complete visualization of the maxillary and mandibular arches, with sufficient sharpness, brightness, and contrast for the morphological evaluation of the permanent first and second molars (1.6, 1.7, 2.6, 2.7, 3.6, 3.7, 4.6, and 4.7). Scans with metallic or motion artifacts that limited interpretation were excluded, as were cases with extensive caries, large restorations, prior endodontic treatment, root fractures, immature apices, extensive bone pathology, or other conditions that prevented a reliable morphological assessment.

The study was reported following the Strengthening the Reporting of Observational Studies in Epidemiology (STROBE) guidelines for observational studies. CBCT scans or teeth with incomplete visualization, severe artifacts, or unidentifiable anatomical landmarks were considered non-evaluable and excluded from the corresponding analysis. For this reason, statistical imputation of missing data was not performed, because only images and teeth that met the minimum criteria for diagnostic evaluability were analyzed.

Initially, 774 CBCT scans were reviewed. After applying the eligibility criteria and confirming diagnostic quality, the final sample consisted of 382 CBCT scans. Within these studies, 692 molars with taurodontism were identified. To ensure a consistent interpretation of the results, two levels of analysis were differentiated: the patient/CBCT level and the tooth level. To estimate prevalence and evaluate its association with patient variables, such as sex and age group, the CBCT scan was considered the unit of analysis, and “presence” was defined as the identification of ≥1 molar with taurodontism in the study. For morphological classification and distribution according to dental arch and tooth type, the taurodontic molar was considered the unit of analysis. The age group was categorized into young adults (18–29 years) and adults (30–59 years). Sex was recorded as male or female based on the data provided in the record associated with the CBCT. Given the retrospective design based on available records, all scans that met the criteria during the study period were included; therefore, no a priori sample size calculation was performed.

### 2.3. Acquisition and Analysis of CBCT Images

The images were acquired using a Newton Giano HR CBCT scanner (Cefla, Imola, Italy; manufactured in 2020), with standardized acquisition parameters of 90 kVp, 3 mA, and an exposure time of 3.6 s. The images were generated with an isotropic voxel size of 230 μm and a field of view (FOV) of 13 × 10 cm. The analysis was performed using multiplanar reconstructions in coronal and sagittal sections, maintaining a slice thickness of 0.23 mm [6,18]. For each permanent molar evaluated, the image was oriented following the longitudinal axis of the tooth, ensuring the alignment of the crown, the pulp chamber, the furcation area, and the longest root in the same reference plane. Measurements were performed on sagittal or coronal sections, depending on which plane allowed for the clearest observation of the vertical extension of the pulp chamber, the chamber floor, the furcation, and the apex of the longest root. In each case, the section where the pulp chamber and its relationship with the cement–enamel junction (CEJ) were most evident was selected, avoiding oblique sections or distorted images that could alter the measurements. Image evaluation and processing were performed on an LG gram Pro (16T90SP) computer, equipped with an Intel Core i7 processor, a screen resolution of 2560 × 1600 pixels, 16 GB of RAM, and the Windows 10 Pro operating system, using the specialized software NNT Viewer version 14.0.1. Image evaluation and measurements were performed by two investigators who had been previously calibrated by a specialist in oral and maxillofacial radiology. Calibration was carried out prior to the main analysis using a sub-sample of 20 CBCT scans that were not part of the final study sample. This sub-sample was selected to include molars with and without taurodontism, as well as cases representative of the different morphological subtypes. During the calibration process, the specialist guided the researchers in identifying anatomical landmarks, multiplanar tooth orientation, measuring the AB, AC, and BD distances, and applying the morphometric criteria for taurodontism classification. Subsequently, the two researchers independently evaluated the same sub-sample, identifying the presence or absence of taurodontism and assigning the corresponding morphological classification.

The inter-examiner agreement was evaluated using the Kappa coefficient, obtaining a value of 0.87. To evaluate intra-examiner agreement, one of the researchers repeated the assessment of the same sub-sample after a 5-day interval, obtaining a Kappa value of 1.00. These values corresponded to the reproducibility of the categorical classification (i.e., presence/absence of taurodontism and morphological subtype) rather than the agreement of continuous measurements. Therefore, the intra-examiner value of 1.00 should be interpreted with caution as categorical reproducibility within the calibration sub-sample. The examiners were not formally blinded to patients’ sex or age during the CBCT assessment, as these variables were available in the retrospective records associated with the imaging database. However, the morphological evaluation was performed using predefined objective morphometric criteria.

In cases of diagnostic doubt, measurements near the cut-off point, or discrepancies among evaluators during the main analysis, the molar was re-evaluated using multiplanar reconstructions, and the final classification was established by consensus. This procedure was particularly relevant for mild or borderline cases, as small measurement differences could modify the morphological classification.

### 2.4. Diagnostic Criteria for Taurodontism

**Figure 1:**
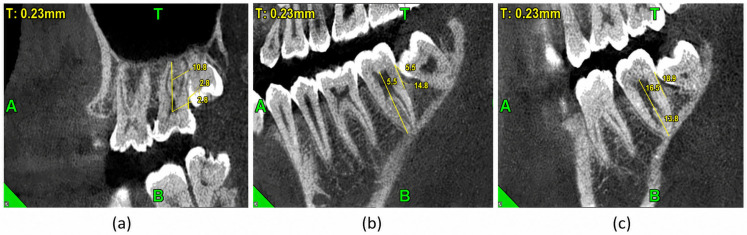
Representative cone-beam computed tomography (CBCT) images of permanent molars with taurodontism, classified according to the taurodontic index (TI). From left to right: (**a**) hypotaurodontism (TI = 25.92%), (**b**) mesotaurodontism (TI = 37.16%), and (**c**) hypertaurodontism (TI = 47.10%). Yellow lines and numeric values indicate the linear measurements used to calculate the TI. The green letters are orientation markers generated by the CBCT viewing software.

**Figure 2:**
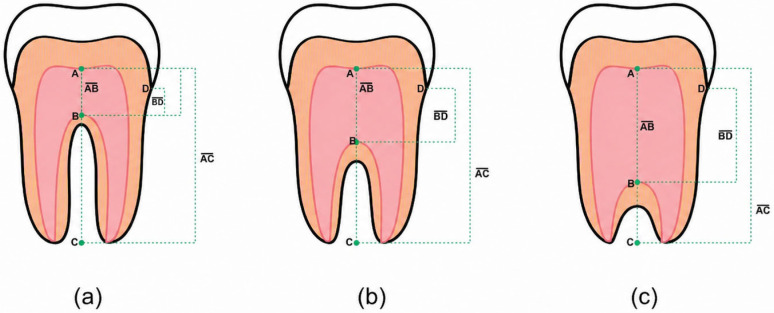
Schematic representation of the classification of taurodontism based on the taurodontic index (TI). (**a**) The subtypes hypotaurodontism (20 ≤ TI < 30), (**b**) mesotaurodontism (30 ≤ TI < 40), and (**c**) hypertaurodontism (40 ≤ TI < 75) are illustrated. AB: distance between the roof and floor of the pulp chamber; AC: distance between the roof of the pulp chamber and the apex of the longest root; BD: vertical distance between the amelocemental junction and the highest point of the floor of the pulp chamber. Colors are used only for schematic visualization.

Taurodontism was identified and classified according to the criteria described by Shifman and Chanannel. The vertical distance between the roof and floor of the pulp chamber (|AB|), the distance between the roof of the pulp chamber and the apex of the longest root (|AC|), and the vertical distance between the amelocemental junction and the highest point of the pulp chamber floor (|BD|) were measured. The AB distance was measured vertically from the highest point of the pulp chamber roof to the highest point of the pulp chamber floor. The AC distance was measured from the same point on the pulp chamber roof to the apex of the longest root. The BD distance was recorded from the cement–enamel junction to the highest point of the pulp chamber floor. When there was uncertainty regarding the exact location of these anatomical points, the adjacent sections were reviewed before establishing the final measurement. Based on the measurements |AB| and |AC|, the taurodontic index (TI) was calculated using the formula TI = (|AB|/|AC|) × 100 [6,19,20] (Figure 1). Taurodontism was considered present when a tooth had a TI > 20 and a |BD| distance > 2.5 mm. According to the TI value, the teeth were classified as hypotaurodontism (20 ≤ TI < 30), mesotaurodontism (30 ≤ TI < 40), and hypertaurodontism (40 ≤ TI < 75) [8,20]. The morphological classification is shown schematically in Figure 2.

### 2.5. Variables and Statistical Analysis

The presence of taurodontism at the CBCT level was defined as the identification of at least one permanent first or second taurodontic molar per scan. The prevalence was calculated at the CBCT/patient level, using the total number of evaluated tomographies as the denominator, and at the tooth level, considering only the permanent first and second molars that were present and evaluable. Tooth-specific prevalence was also estimated based on the number of taurodontic molars and evaluable teeth in each position. Categorical variables were described using absolute and relative frequencies. The bivariable association between CBCT-level taurodontism and sex or age group was evaluated using Pearson’s chi-square test and crude odds ratios with 95% CIs. Subsequently, multivariable binary logistic regression was applied to obtain odds ratios adjusted for sex, age group, and the number of evaluable molars per CBCT. The analyses by tooth type, morphological subtype, and dental arch were descriptive. Additionally, a sensitivity analysis was performed by excluding mild hypotaurodontism cases to estimate the restricted prevalence of moderate/severe forms. The significance level was set at *p* < 0.05. All analyses were performed using IBM SPSS Statistics for Windows, version 30.0.3.

## 3. Results

### 3.1. Prevalence of Molar Taurodontism at the CBCT and Dental Level

Table 1 shows the prevalence of molar taurodontism according to the unit of analysis. Dental prevalence was calculated using the first and second permanent molars present and evaluable as the denominator. Of the 2472 evaluable molars, 692 were taurodontic (28.0%) and 1780 were non-taurodontic (72.0%). At the CBCT level, 266 of 382 tomographies showed at least one taurodontic molar, representing a prevalence of 69.6%.

### 3.2. Distribution of Evaluable and Non-Evaluable Molars According to Age Group

Table 2 shows the distribution of evaluable, non-evaluable, and taurodontic molars according to age group. Young adults aged 18–29 presented 1285 evaluable molars, 203 excluded/non-evaluable molars, and 429 taurodontic molars, whereas adults aged 30–59 presented 1187 evaluable molars, 381 excluded/non-evaluable molars, and 263 taurodontic molars. Due to this difference in the number of evaluable molars between groups, this variable was included as a covariate in the multivariate analysis.

### 3.3. Patient-Level Factors Associated with Taurodontism 

Table 3 shows that, in the bivariate analysis, taurodontism on CBCT was more frequent in females than in males and in young adults aged 18–29 years than in adults aged 30–59 years. In the multivariable binary logistic regression model, adjusted for sex, age group, and number of CBCT-evaluable molars, female sex maintained higher odds of presenting with taurodontism on CBCT (adjusted OR = 1.73; 95% CI: 1.10–2.73; *p* = 0.018). Likewise, young adults presented higher odds than adults aged 30–59 years (adjusted OR = 2.34; 95% CI: 1.45–3.77; *p* < 0.001). The number of CBCT-evaluable molars showed no statistically significant association (adjusted OR = 1.13; 95% CI: 0.97–1.32; *p* = 0.112).

### 3.4. Distribution of Molar Taurodontism According to Tooth and Morphological Classification

Table 4 shows that, at the dental level, 692 taurodontic molars were identified. The highest frequency corresponded to tooth 27 (139/692; 20.1%), followed by teeth 17 (130/692; 18.8%) and 16 (125/692; 18.1%). According to the morphological classification, hypotaurodontism predominated (594/692; 85.8%), followed by mesotaurodontism (96/692; 13.9%) and hypertaurodontism (2/692; 0.3%).

### 3.5. Dental Prevalence of Molar Taurodontism Using Tooth-Specific Denominators

Table 5 shows the tooth-specific prevalence of molar taurodontism using tooth-specific denominators. The total number of potential teeth was estimated from 382 CBCT scans, considering permanent first and second molars. Non-evaluable teeth corresponded to missing molars or teeth that did not meet the selection criteria. Tooth-specific prevalence was calculated by dividing the number of taurodontic molars by the evaluable teeth for each tooth position. The highest prevalence was observed in tooth 27 (139/329; 42.2%), followed by teeth 16 (125/304; 41.1%) and 17 (130/318; 40.9%). Maxillary molars presented the highest prevalences, whereas mandibular molars showed lower values, particularly teeth 46 (11/292; 3.8%) and 36 (18/289; 6.2%).

### 3.6. Distribution by Dental Arch

Table 6 shows that, of the 692 taurodontic molars identified, 502 (72.5%) were located in the maxillary arch and 190 (27.5%) in the mandibular arch. These values indicate that taurodontic molars were observed more frequently in the maxilla. However, this distribution should not be interpreted as arch-specific prevalence, because the denominator corresponded to the total number of taurodontic molars and not to the total number of evaluable molars in each arch.

### 3.7. Sensitivity Analysis of the Prevalence of Taurodontism Based on Diagnostic Severity in CBCT Tomographies

Table 7 presents the sensitivity analysis of taurodontism prevalence on CBCT scans after excluding mild cases of hypotaurodontism. When considering only moderate/severe forms, 67 of 382 CBCT scans showed at least one molar with mesotaurodontism or hypertaurodontism, representing a restricted prevalence of 17.5%. This finding indicates that the overall prevalence of 69.6% was primarily influenced by the detection of mild forms using CBCT.

## 4. Discussion

Taurodontism carries clinical relevance because it alters the internal anatomy of the tooth and can complicate the planning of endodontic, restorative, and surgical procedures [21]. Therefore, determining its prevalence and presentation patterns using CBCT holds descriptive and epidemiological value [22,23]. Nonetheless, the findings of the present study must be interpreted within its specific methodological scope: the evaluation was limited to permanent first and second molars in a selected Peruvian CBCT-based sample, rather than the entire dentition or a representative sample of the Peruvian population. In this study, the overall prevalence of taurodontism was high at the CBCT level, as 69.6% of the tomographies showed at least one taurodontic molar. However, this percentage must be interpreted specifically as patient/CBCT-level prevalence, defined by the presence of ≥1 permanent first or second taurodontic molar on the scan, and not as the proportion of taurodontic molars or as a general prevalence of the Peruvian population. This distinction is important because each CBCT could include up to eight evaluable molars, and the presence of a single taurodontic molar classified the scan as positive. Consequently, the 69.6% value naturally tends to be higher than the prevalence calculated at the tooth level. Indeed, when considering only the permanent first and second molars that were present, evaluable, and met the selection criteria, 692 out of 2472 molars were classified as taurodontic, corresponding to a tooth-level prevalence of 28.0%. This differentiation between CBCT/patient-level prevalence and tooth-level prevalence enhances the epidemiological interpretation of the results and prevents inappropriate direct comparisons with studies that evaluated fewer teeth, utilized panoramic radiographs, applied different diagnostic thresholds, or analyzed full dentitions.

This finding is relevant for the analyzed Peruvian sample, where CBCT-based evidence remains scarce and much of the available information stems from Asian or Middle Eastern populations [7,24]. Although the observed prevalence was higher than that reported in China, Saudi Arabia, and Iran [7,8,14], this difference should not be attributed solely to potential biological variations among populations. Recent studies have also reported relatively high prevalences, though lower than that observed in the present study, such as 34% in a contemporary Polish population and 33.6% per patient in Israel. Furthermore, a recent review estimated an average prevalence of 11.8%, with an extremely wide range among studies, from 0.1% to 48%, evidencing a marked heterogeneity in the literature. These variations are likely influenced by methodological differences, given that the prevalence of taurodontism can vary according to the diagnostic method, the sensitivity of the criteria employed, the effects of age and tooth wear on pulp morphology, as well as the type and number of teeth included in the analysis [6,19]. In the present study, the high prevalence appears to be explained not only by potential sample characteristics but also by the use of CBCT, the systematic inclusion of permanent first and second molars, and the predominance of mild forms, especially hypotaurodontism, which may go unnoticed in less sensitive evaluations [25,26]. In line with this interpretation, the sensitivity analysis showed that when mild cases of hypotaurodontism were excluded and only mesotaurodontism and hypertaurodontism were considered, the CBCT-level prevalence decreased from 69.6% to 17.5%. This result indicates that the elevated overall prevalence was driven primarily by mild forms detected via CBCT, rather than a high frequency of moderate or severe forms.

Taken together, the observed magnitude appears to depend not only on the studied population, but also on the analytical approach, imaging modality, diagnostic thresholds used, and the unit of analysis considered. Therefore, the interpretation of these results must be approached with caution, recognizing that the study provides descriptive epidemiological evidence from a selected Peruvian CBCT-based sample, rather than generalizable estimates for the Peruvian population as a whole.

Regarding sex, taurodontism detected at the CBCT level was more frequent in females than in males. This association remained after adjusting for age group and the number of evaluable molars in the multivariable binary logistic regression model. However, this finding should not be overinterpreted, as the magnitude of the association was modest and the confidence interval of the crude OR was close to the null value. Therefore, it should not be assumed as evidence of a robust biological pattern associated with sex, but rather as an association observed within this selected Peruvian CBCT-based sample. This pattern is partially consistent with that described by Aydın et al., who observed a higher prevalence of taurodontism in females [17]. Likewise, Arıcıoğlu et al. reported differences by sex in a CBCT-based evaluation (*p* < 0.05) [27]. However, not all studies are consistent on this point. Li et al. found a virtually similar distribution between males and females [7], demonstrating that the available findings remain heterogeneous. This lack of uniformity suggests that the association with sex should not be considered universal, as it could depend on the characteristics of each sample, the source population, the study design, the diagnostic criteria used, and the type of statistical analysis applied. Consequently, although the observed finding is epidemiologically relevant within this sample, it must be interpreted with prudence and requires confirmation in multicenter studies with larger, representative samples to determine whether a consistent association exists between sex and molar taurodontism.

Regarding age, taurodontism was detected more frequently in the 18–29 age group than in the 30–59 age group. This association remained significant after adjusting for sex and the number of evaluable molars in the multivariable binary logistic regression model. However, this finding should be interpreted as a difference in taurodontism detected via CBCT and not necessarily as a higher true biological prevalence in young adults. This result is partially consistent with the findings of Jabali et al., who also reported a higher prevalence in younger age groups [14]. Nonetheless, the available evidence regarding the relationship between age and taurodontism remains inconsistent. In the present study, young adults presented a higher number of evaluable molars than adults aged 30–59, whereas the adult group presented more excluded or non-evaluable molars. This difference can influence the probability of classifying a CBCT scan as positive under the criterion of the presence of ≥1 taurodontic molar, given that a lower number of evaluable molars reduces the opportunity to detect at least one molar with taurodontism.

Furthermore, the identification of taurodontism can be influenced by age-related changes and tooth wear. Several recent CBCT-based studies have excluded teeth with crowns, caries, restorations, or prior treatments, because these conditions can affect pulp chamber morphology and diagnostic accuracy [6,8,19,28]. In older individuals, the progressive deposition of secondary or tertiary dentin, the reduction in pulp chamber space, tooth wear, restorations, caries, or prior treatments can reduce the diagnostic visibility of taurodontic morphology on CBCT. Therefore, the observed association with age should be interpreted as a possible difference in the imaging detectability of taurodontism—potentially influenced by the availability and visibility of evaluable molars—and not as definitive evidence of a causal biological difference in the true prevalence of taurodontism. The distribution by tooth type and morphological classification constitutes one of the most relevant descriptive findings of the study. Of the 692 identified taurodontic molars, the highest frequencies were observed in teeth 27, 17, and 16. Hypotaurodontism accounted for 594/692 cases, equivalent to 85.8%; mesotaurodontism accounted for 96/692 cases, equivalent to 13.9%; and hypertaurodontism accounted for 2/692 cases, equivalent to 0.3%. This pattern confirms that the majority of cases corresponded to mild forms of taurodontism, whereas severe forms were infrequent.

This pattern is consistent with previous CBCT-based studies, which have described a predominance of hypotaurodontism and a greater involvement of posterior molars. Li et al. reported that the maxilla presented a higher incidence of taurodontism and that the maxillary second molar was the most frequently affected tooth [7]; similarly, Jabali et al. and Davvaz et al. also identified hypotaurodontism as the most common presentation [8,14]. Likewise, Arıcıoğlu et al. and Akbarizadeh et al. reported a higher frequency in second molars among the evaluated teeth [27,29], and Da et al. observed that the frequency increased toward the more posterior teeth of the dental arch, supporting the distribution pattern found [19]. From a diagnostic perspective, this finding suggests that the most common forms do not necessarily correspond to extreme cases, but rather to mild morphological variants that could go unnoticed if the internal anatomy of the tooth is not carefully evaluated [28,30]. The higher proportion of taurodontic molars in the maxilla represents a descriptive pattern observed within this sample. Jabali et al. reported a higher frequency in maxillary molars than in mandibular molars [14], and Aydın et al. also observed a higher occurrence in upper molars, especially in second molars [17]. Consistently, a greater distribution of taurodontic molars in the maxillary arch was observed in the present study. However, this result should not be interpreted as a causal explanation for why the maxilla would present a higher frequency, nor as arch-specific prevalence, because the denominator used corresponded to the total number of identified taurodontic molars and not to the total number of evaluable molars in each arch. Therefore, this finding should be understood as a descriptive anatomical distribution among the molars already classified as taurodontic.

Among the main limitations of the study are its retrospective, observational, and cross-sectional design, as well as its single-center nature, which limits the possibility of directly extrapolating the findings to the entire Peruvian population. Additionally, the possibility of selection bias must be considered, because the sample originated from a private radiologic center and consisted of patients referred for CBCT imaging between 2022 and 2024. Therefore, this sample should not be interpreted as population-based, since patients who require CBCT may differ from the general population regarding dental status, presence of missing teeth, restorative or endodontic history, pathologies, surgical or orthodontic needs, and other diagnostic indications. Although the initial and final numbers of evaluated CBCT scans were recorded, the specific reasons for exclusion were not documented systematically or in a mutually exclusive manner for each excluded scan. Consequently, it was not possible to provide a quantified breakdown of the reasons for exclusion. This should be considered a limitation inherent to the retrospective image selection process.

Likewise, the specific clinical indications for referral were not available in a standardized manner within the database, and therefore could not be analyzed. Compounding this, the study included only CBCT scans with adequate diagnostic quality and molars that were present, evaluable, and free of conditions that could alter the morphology of the pulp chamber or roots. The exclusion of teeth with extensive restorations, advanced caries, previous endodontic treatment, fractures, immature apices, or other alterations was necessary to ensure a reliable morphological evaluation; however, it may have also resulted in a highly selected sample. Consequently, the results must be interpreted as descriptive epidemiological evidence from a selected Peruvian CBCT-based sample, rather than generalizable estimates for the Peruvian population as a whole.

Another relevant limitation is the potential diagnostic overestimation associated with the inclusion of mild or borderline cases, especially those classified as hypotaurodontism. Although established morphometric criteria were applied, the use of CBCT can increase the detection of subtle anatomical variations that might not be identified through two-dimensional radiographs or via stricter diagnostic criteria. To address this aspect, a sensitivity analysis was performed by excluding hypotaurodontism cases, which allowed for a prevalence estimation considering only moderate and severe forms.

It should also be noted that age-related changes in the pulp chamber could have influenced the detection of taurodontism. The progressive deposition of secondary or tertiary dentin and the reduction in pulp space in older individuals could render the taurodontic morphology less evident on CBCT. Therefore, the observed associations with age must be interpreted as potential differences in the diagnostic visibility of taurodontism via CBCT, rather than evidence of causal biological differences in the true prevalence of this anatomical variation. Despite these limitations, the study possesses important strengths, such as the exclusive use of CBCT, the application of objective morphometric diagnostic criteria, and the evaluation of a large sample of permanent first and second molars. CBCT allowed for a more precise three-dimensional assessment of pulp and root morphology, which is particularly useful for describing anatomical variations such as taurodontism. From an epidemiological perspective, the findings provide local evidence regarding the frequency, tooth distribution, and morphological patterns of molar taurodontism in a Peruvian sample evaluated using CBCT. Nonetheless, the associations observed in females, young adults, and maxillary molars should be interpreted as descriptive findings within this selected sample, and not as definitive causal or biological differences.

In the Peruvian context, where imaging studies on dental anatomical variations remain scarce, this research contributes to improving the epidemiological understanding of molar taurodontism. However, multicenter studies with larger, representative samples and available information about the clinical indications for CBCT referral are required to confirm these patterns in Peru and Latin America.

## 5. Conclusions

Molar taurodontism, defined according to the criteria of this study as the presence of at least one taurodontic permanent first or second molar, showed a high prevalence at the patient/CBCT level in this CBCT-based Peruvian sample. However, the prevalence at the tooth level was lower, highlighting the importance of differentiating between estimates made at the patient/CBCT level and those calculated at the tooth level.

Among taurodontic molars, most were located in the maxillary arch, with hypotaurodontism being the predominant subtype. However, these associations should be interpreted with caution. The association with sex was modest and requires confirmation in larger studies, whereas the association with age may be influenced by the greater diagnostic visibility of the pulp chamber in young adults and by age-related pulp changes, rather than by a real biological difference in prevalence.

## Figures and Tables

**Table 1:** Prevalence of molar taurodontism according to the unit of analysis.

Indicator	n/N	%
CBCT/patient prevalence: ≥1 taurodontic molar	266/382	69.6
Prevalence at the dental level: taurodontic molars among evaluable molars	692/2472	28.0
Evaluable molars without taurodontism	1780/2472	72.0

**Table 2:** Distribution of evaluable, non-evaluable, and taurodontic molars according to age group.

Age Group	CBCT n	Evaluable Molars n	Excluded/Non-Evaluable Molars n	Taurodontic Molars n
Young adults aged 18–29 years	186	1285	203	429
Adults aged 30–59 years	196	1187	381	263
Total	382	2472	584	692

**Table 3:** Crude and adjusted association between patient variables and taurodontism at the CBCT level.

Variable	Presence n (%)	Absence n (%)	C-OR (95% CI)	*p*-Value	A-OR (95% CI)	*p*-Value
Sex						
Male	117 (64.6)	64 (35.4)	1 (reference)	—	1 (reference)	—
Female	149 (74.1)	52 (25.9)	1.57 (1.01–2.43)	0.044	1.73 (1.10–2.73)	0.018
Age group						
Adults, 30–59 years	119 (60.7)	77 (39.3)	1 (reference)	—	1 (reference)	—
Young adults, 18–29 years	147 (79.0)	39 (21.0)	2.44 (1.55–3.84)	<0.001	2.34 (1.45–3.77)	<0.001
Number of evaluable molars	—	—	—	—	1.13 (0.97–1.32)	0.112

C-OR: crude odds ratio; A-OR: adjusted odds ratio; CI: confidence interval.

**Table 4:** Distribution of taurodontic molars according to tooth and morphological subtype.

Tooth	Hypo n (%)	Meso n (%)	Hyper n (%)	Total n (%)
16	111 (88.8)	14 (11.2)	0 (0.0)	125 (18.1)
17	113 (86.9)	17 (13.1)	0 (0.0)	130 (18.8)
26	98 (90.7)	10 (9.3)	0 (0.0)	108 (15.6)
27	115 (82.7)	23 (16.5)	1 (0.7)	139 (20.1)
36	17 (94.4)	1 (5.6)	0 (0.0)	18 (2.6)
37	65 (80.2)	16 (19.8)	0 (0.0)	81 (11.7)
46	10 (90.9)	1 (9.1)	0 (0.0)	11 (1.6)
47	65 (81.2)	14 (17.5)	1 (1.2)	80 (11.6)
Total	594 (85.8)	96 (13.9)	2 (0.3)	692 (100.0)

Hypo: Hypotaurodontism; Meso: Mesotaurodontism; Hyper: Hypertaurodontism.

**Table 5:** Prevalence of molar taurodontism using tooth-specific denominators.

Molar	Total Possible	Evaluable	Excluded/Non Evaluable	Normal n	Hypo n	Meso n	Hyper n	Taurodontic n	Dental Prevalence %
16	382	304	78	179	111	14	0	125	41.1
17	382	318	64	188	113	17	0	130	40.9
26	382	297	85	189	98	10	0	108	36.4
27	382	329	53	190	115	23	1	139	42.2
36	382	289	93	271	17	1	0	18	6.2
37	382	321	61	240	65	16	0	81	25.2
46	382	292	90	281	10	1	0	11	3.8
47	382	322	60	242	65	14	1	80	24.8
Total	3056	2472	584	1780	594	96	2	692	28.0

Hypo: Hypotaurodontism; Meso: Mesotaurodontism; Hyper: Hypertaurodontism.

**Table 6:** Distribution of taurodontic molars according to dental arch.

Arch	Included Teeth	n	%
Maxillary	16, 17, 26, 27	502	72.5
Mandibular	36, 37, 46, 47	190	27.5
Total	—	692	100.0

**Table 7:** Sensitivity analysis of the prevalence of taurodontism according to diagnostic severity in CBCT tomographies.

Diagnostic Criterion	CBCT with Taurodontism n/N	Prevalence %
Any form of taurodontism: hypotaurodontism + mesotaurodontism + hypertaurodontism	266/382	69.6
Moderate/severe taurodontism: mesotaurodontism + hypertaurodontism	67/382	17.5
CBCT with at least one case of mesotaurodontism	66/382	17.3
CBCT with at least one case of hypertaurodontism	2/382	0.5

These categories are not mutually exclusive at the CBCT level, since a single scan may show at least one molar with mesotaurodontism and at least one molar with hypertaurodontism.

## Data Availability

The anonymized dataset supporting the findings of this study is provided as Supplementary Materials. The original CBCT images are not publicly available due to privacy, ethical, and legal restrictions related to patients’ idmaging records.

## Supplementary Materials

The following supporting information can be downloaded at: https://www.mdpi.com/article/10.3390/dj14060357/s1, Table S1: Study dataset.
